# The Lived Experience of Adolescents With Type 1 Diabetes Mellitus in Bahir Dar, Ethiopia: Phenomenological Study

**DOI:** 10.2196/71179

**Published:** 2026-08-05

**Authors:** Yassin Hussin, Desalegn Awle, Hailemariam Mekonnen Workie, Desalegne Amare

**Affiliations:** 1 School of Nursing and Midwifery, College of Health and Medical Science, Haramaya University, P.O. Box 138, Dire Dawa, Ethiopia, 25 1925449257; 2 College of Medicine and Health Science, Bahir Dar University, Bahir Dar, Ethiopia

**Keywords:** adolescents, adolescent health, Bahir Dar, chronic illness, Ethiopia, lived experience, type 1 diabetes mellitus

## Abstract

**Background:**

Type 1 diabetes mellitus (T1DM) is one of the most common chronic diseases among adolescents and is posing a threat to health and potentially endangering life. It can have a significant impact on the physical, social, and emotional development of adolescents. Understanding the lived experiences of adolescents with T1DM is crucial for improving their health outcomes and future aspirations. However, there is currently limited research, and existing studies on psychosocial and self-care perspectives do not highlight their lived experiences in Ethiopia.

**Objective:**

This study aimed to explore the lived experiences of adolescents living with T1DM in Bahir Dar, Ethiopia.

**Methods:**

A descriptive phenomenological design was used. The study was conducted at Felege Hiwot Comprehensive Specialized Hospital in Bahir Dar from March to May 2023. Ten adolescents aged 10‐19 years receiving follow-up care at the hospital were selected using purposive sampling. Data were collected through face-to-face, in-depth interviews using a semistructured guide translated into Amharic. All interviews were conducted by the principal investigator, audio-recorded, transcribed verbatim, and translated conceptually. Thematic analysis was conducted using ATLAS.ti (8.4.24; ATLAS.ti Scientific Software Development GmbH) software, guided by the Colaizzi phenomenological method.

**Results:**

The study included 10 participants (7 female and 3 male), aged 13‐18 years, with the duration of living with T1DM ranging from 6 months to 11 years. It identified 4 key themes in adolescents managing insulin dependence. First, psychoemotional factors were significant, with participants experiencing stress, anxiety, low self-esteem, and negative emotions such as fear and hopelessness, although some also reported positive feelings from strong social support. Second, supportive factors were crucial, as family, friends, teachers, health care providers, and community health insurance offered essential emotional and practical help. Third, significant challenges included insulin unavailability, financial issues, fasting-related nonadherence, fear of hypoglycemia, and sleep disturbances, all impacting their well-being. Finally, adolescents used various coping strategies, such as avoidance, acceptance, prayer, and social engagement, to manage emotional distress and maintain daily functioning.

**Conclusions:**

Adolescents with T1DM in Bahir Dar face multifaceted challenges that affect their emotional well-being and diabetes management. Despite these difficulties, they demonstrate resilience through various coping strategies and benefit from strong support systems. These findings underscore the need for adolescent-centered diabetes care, improved insulin access, and psychosocial support in both clinical and community settings.

## Introduction

### Background

Diabetes mellitus (DM) is a metabolic disorder that causes chronic hyperglycemia due to abnormalities in insulin secretion or action [1]. This condition leads to pathophysiologic changes in multiple organ systems, with 4 main categories, including type 1 DM (T1DM), type 2 DM, gestational DM, and specific types resulting from other causes [2]. T1DM, previously known as insulin-dependent diabetes, is a common chronic childhood disease with peak diagnosis in the midteens [3]. Its exact cause is unknown. T1DM results from autoimmune β-cell destruction, usually leading to absolute insulin deficiency, including latent autoimmune diabetes in adults [4,5].

Adolescence is a transitional phase between childhood and adulthood that involves physical, cognitive, social, and emotional changes leading toward independence [6]. The World Health Organization (WHO) defines adolescents as individuals aged 10-19 years. [7].

In the past 20 years, the global prevalence of diabetes has nearly tripled, with a significant increase in children and adolescents living with the disease [8]. Currently, there are approximately 50,600 children and adolescents under the age of 20 living with T1DM in Africa [9]. Ethiopia ranks fourth in the number of people with diabetes [8], and the estimated incidence of T1DM among children and adolescents is 2.4 per 100,000 per year [10].

T1DM in adolescents has negative impacts on their daily activities, personal aspirations, and psychological well-being [11]. It can lead to embarrassment, discrimination, limited social relationships, poorer diabetes management, cognitive decline, and a higher risk of developing cardiovascular problems [12]. The unique developmental nature of adolescence, including physical, cognitive, and psychosocial changes, complicates diabetes management for this population. Therefore, achieving ideal metabolic control can be challenging while balancing their developmental needs [13].

Adolescents with T1DM view their condition as a challenge and strive to lead normal lives [14]. Some hope for a cure [15], while others feel different because they have to follow a care plan [16]. Perceptions of T1DM in adolescents tend to be negative [17].

According to a qualitative study, teenagers with T1DM report feeling anxious about blood tests and the long-term implications of their condition. Some of them also expressed discomfort or embarrassment about injecting insulin [18].

Children with T1DM tend to experience more sleep problems, which can impact their behavioral and executive functioning [19,20]. The American Diabetes Association’s Standards now recognize the importance of evaluating sleep in clinical settings, as there is emerging evidence showing a relationship between sleep quality and glycemic control [9].

Studies have shown that both family and peer support are important for adolescents with T1DM to effectively manage their condition and adjust to living with a chronic illness. Peer support has been effectively linked to improved compliance and better overall outcomes [21]. In addition, research has shown that adolescents with well-controlled diabetes tend to have stronger family bonds and experience less conflict [22]. Improving diabetes care and support within school settings has been shown to enhance diabetes management and quality of life for this population [23]. However, another study conducted in Ethiopia uncovered that some adolescents faced difficult family dynamics, such as excessive parental control and constant warnings about diabetes management, which created tension [24,25]. Another qualitative study revealed that acceptance, avoidance, and adaptive coping strategies were commonly used by adolescents with T1DM [26].

While there is some research on the lived experiences of individuals with T1DM in Ethiopia, such as studies conducted in North Eastern Ethiopia examining psychosocial and economic perspectives [27] and self-care practices [28], there is still a gap in understanding the specific lived experiences of adolescents with T1DM. This study aimed to fill this gap by exploring the unique experiences of adolescents living with T1DM in this region. The findings are essential for providing a platform for participants to voice their concerns and needs, ultimately leading to improved care and quality of life for both themselves and future adolescents with T1DM. Additionally, it will assist program developers in formulating strategies tailored to adolescents living with T1DM.

### Objective

The objective of this study was to explore the lived experiences of adolescents living with T1DM in Bahir Dar, Ethiopia, focusing on their emotional, social, and daily life experiences while managing the disease.

## Methods

### Study Setting and Period

The study was conducted at Felege Hiwot Comprehensive Specialized Hospital (FHCSH), located in Bahir Dar City, Amhara region, Ethiopia. The hospital was established during the regime of Emperor Haileselassie in April 1963 and provides services for neighboring regions, including Benishangul-Gumuz, serving more than 10 million people.

The department of internal medicine at FHCSH has both inpatient and outpatient units. The hospital also has outpatient follow-up clinics for DM. The chronic follow-up clinic was staffed with internists, residents, general practitioners, and a nurse. Follow-up clients for the diabetes clinic were scheduled for Monday and Tuesday but could also be seen on other days depending on their appointments. On average monthly, 500‐600 patients with chronic conditions were seen. In addition, 67 children were being followed up for diabetes at the time of data collection. The study was conducted from March to May 2023.

### Study Design and Characteristics of Participants

This study used a descriptive phenomenological design to explore the lived experiences of adolescents with T1DM.

### Participant Selection and Sampling Strategy

The total number of participants was 10. Participants were selected using purposive sampling, guided by specific inclusion criteria to ensure rich, relevant data. Eligible participants were adolescents aged 10‐19 years, diagnosed with T1DM for at least 6 months, receiving follow-up care at FHCSH, and able to communicate their experiences. Adolescents with cognitive impairments or acute medical conditions that could interfere with the interview process were excluded. Potential participants were initially identified through the FHCSH diabetes clinic registry, which tracks known patients with diabetes who attend regularly scheduled monthly follow-up appointments. When an identified, eligible adolescent arrived at the hospital for their routine monthly visit, they were contacted and directly approached in person by the principal investigator (PI). The PI introduced the study, explained its purpose, and screened the adolescents against the eligibility criteria. Enrollment was finalized on-site after obtaining written informed consent (and assent from the minor, where applicable) prior to conducting the interviews. Recruitment continued until data saturation was achieved, defined as the point at which no new themes or insights emerged from subsequent interviews.

### Data Collection Method and Procedures

The data were collected through face-to-face in-depth interviews using semistructured, open-ended, guiding questions. The interview guide was developed in English, which comprised sociodemographic, psychological, social, emotional, and economic impact (Multimedia Appendix 1). The question guide translated into Amharic for better understanding. Participants were assigned study codes (P1, P2, P3, etc) based on the order of interviewing. The average interview time ranged from 30 to 60 minutes and included questions about the patient’s sociodemographic background and experiences with T1DM as adolescents.

The PI conducted all the interviews. Participants who came for follow-up, communicated with nurses before or after they received treatment in the follow-up room. Meetings were scheduled at times convenient for participants once they agreed to participate in the study. All interviews, except for one, took place at the participant’s home.

At the beginning of each interview, the researcher and assistant introduced themselves and explained the study’s objectives. Participants were informed that their participation was voluntary and that they could decline or withdraw at any time. The interviews began after participants provided written informed assent (and parental consent, where applicable), completed a brief demographic questionnaire, indicated that they were comfortable proceeding, and granted permission for the interview to be audio-recorded for verbatim transcription. The PI interviewed one adolescent every 3 days, and during the interview, no participant showed signs of distress, and everyone continued to talk in a relaxed manner. The audio recorder was checked to ensure uninterrupted recording.

Upon completion of the questions, the PI asked if there was anything else the participant wanted to discuss. The interviewer then thanked the participant and arranged for follow-up contact to ensure accurate transcription.

### Trustworthiness

To ensure the quality of the research findings, the investigators considered different sets of criteria focusing on the credibility, dependability, transferability, and confirmability of the study using various techniques. Prior to data collection, the investigators familiarized themselves with the study setting and established good relationships with relevant staff members. They spent significant time on-site and engaged extensively with the participants. The PI conducted comprehensive field notes, audio recordings, and verbatim transcriptions, which were then reviewed by the coauthor (DA). Additionally, some participants were asked to review the extracted codes to ensure alignment with their experiences and perspectives. The data were described in detail, considering the context of the setting and the purpose of the study. The methods used to procure the data were clearly outlined. Direct quotes from the data were used to validate the findings. A concise summary of the major findings and their implications within the field was provided, with extensive consideration given to limitations and potential areas for future research. The authors sent all collected data tools, raw data, codes generated during the analysis phase, and drawn inferences to researchers not involved in the study for an external audit. The authors securely stored the data. The study ensured that reflexivity and investigators’ own opinions did not affect the data and its interpretation. By reviewing transcript data precisely, comparing codes with the raw data, and checking the findings with the participants’ views several times, the research process was confirmed by the coauthors.

### Researchers’ Position

The researchers involved in this study have no personal connection to the phenomenon being studied (T1DM). However, they all come from health care backgrounds and possess a minimum of a master’s degree in pediatrics and child health nursing. The authors have a high level of experience in qualitative research, although the extent of their experience varies among researchers.

### Data Management and Analysis

Data management and analysis occurred iteratively, commencing immediately following the initial interview to allow preliminary insights to inform subsequent data collection. Audio recordings were transcribed verbatim in Amharic by the PI and translated into English for analysis; back-translation was performed on a subset of transcripts by an independent language expert to ensure semantic equivalence. To guarantee the reproducibility of the study, qualitative data analysis was systematically conducted using ATLAS.ti (version 8.4.24; ATLAS.ti Scientific Software Development GmbH) software, strictly adhering to Colaizzi 7-step descriptive phenomenological framework.

In the first step, the PI repeatedly read the transcripts while listening to the audio files to immerse themselves in the data and gain an intimate understanding of the participants’ contexts. Second, the PI reviewed the transcripts line-by-line within ATLAS.ti to isolate phrases and sentences directly pertaining to the adolescents’ experiences of living with T1DM. Third, the PI articulated the underlying psychological meanings of each significant statement, abstracting the raw data into conceptual insights without stripping away the participant’s original context. Fourth, these formulated meanings were grouped into clusters of themes and subsequent subthemes, which were continuously cross-referenced with the original transcripts to ensure validation. Fifth, the PI integrated the clustered themes into a comprehensive, detailed narrative that fully described the lived experience of adolescent diabetes management. Sixth, this exhaustive description was synthesized and condensed into a succinct statement capturing the essential structure (the “essence”) of the phenomenon. Seventh, member-checking was performed by sharing the preliminary summary of findings with a subset of the participants during their subsequent monthly hospital follow-up visits to ensure the final analysis accurately reflected their true lived realities.

Methodologically, the entire analysis was rigorously anchored in Husserlian descriptive phenomenology, which demands capturing the phenomenon as purely experienced by the participants. To achieve this and eliminate researcher bias, the PI actively practiced Husserlian phenomenological bracketing (epoche) throughout the study. Before and during the analysis, the PI explicitly identified and set aside their own preexisting clinical assumptions, biases, and emotional responses stemming from their professional experience in health care. This bracketing was operationalized by maintaining a detailed reflexive journal, where the PI documented personal reflections after each interview. Furthermore, to enhance trustworthiness, the initial coding and thematic structure generated by the PI were independently reviewed and verified by a coinvestigator experienced in qualitative methods; any coding discrepancies were resolved through collaborative peer debriefing until a 100% consensus on the final thematic framework was reached.

### Ethical Considerations

This study was reviewed and approved by the Institutional Review Board (IRB) of Bahir Dar University, College of Medicine and Health Sciences (approval number: BDU/IRB/2023/041). The research involved human subjects and adhered to ethical principles outlined in the Declaration of Helsinki. A permission letter was obtained from FHCSH. Informed assent and consent were obtained from all participants and their legal guardians prior to data collection. Participants were informed about the study’s purpose, procedures, potential risks, and their right to withdraw at any time without penalty. Consent included permission to audio-record interviews for transcription and analysis. No secondary data analysis was conducted; all data were collected directly from participants for this study. To ensure privacy and confidentiality, all interview data were anonymized during transcription. Participants were assigned codes (eg, P1 or P2) instead of using personal identifiers. Audio recordings, transcripts, and field notes were securely stored and accessible only to the research team. Data were reported in aggregate form, and direct quotes were deidentified to prevent recognition. No financial compensation was provided to participants. However, interviews were scheduled at times and locations convenient to them, and refreshments were offered when appropriate to ensure comfort and respect for their time.

## Results

### Sociodemographic Characteristics of the Participants

A total of 10 adolescents living with T1DM participated in this study, including 7 females and 3 males. The participants were assigned codes from P1 to P10. Participants were aged 10-19 years. Seven of them resided in urban areas, while 3 lived in rural areas. Their educational attainment ranged from primary school to preparatory school. The participants had been living with DM for periods ranging from 6 months to 11 years (Table 1).

**Table 1.** Background characteristics of the participants of the research of lived experience of adolescents living with type 1 diabetes mellitus (T1DM), Bahir Dar, 2023.

Code	Age (years)	Educational status	Health insurance
P1	17	Grade 11	Yes
P2	15	Grade 7	Yes
P3	16	Grade 9	Yes
P4	18	Grade 12	Yes
P5	16	Grade 9	No
P6	13	Grade 6	Yes
P7	17	Grade 12	Yes
P8	16	Grade 9	Yes
P9	18	Grade 8	No
P10	15	Grade 9	Yes

### The Essence of the Lived Experience

The overarching essence of the lived experience for adolescents navigating T1DM within this context is characterized by a fragile, continuous negotiation between an encroaching medical identity and the developmental pursuit of adolescent normality. Rather than experiencing their condition as an isolated medical routine, these participants experienced a profound disruption of self-concept, frequently defining themselves primarily as “patients” burdened by feelings of inferiority, body shame, and existential questioning. This vulnerability is deeply exacerbated by systemic and environmental precarity, including severe sleep disruptions and localized structural threats like the absolute unavailability of insulin. To survive this reality, the adolescent experience is not passive; it is an active, multidimensional struggle for equilibrium. Youth leverage a complex matrix of structural safety nets (eg, Community-Based Health Insurance [CBHI]) and intimate social ecosystems (family, school, and peers) to counterbalance their endurance. Ultimately, their lived experience is defined by a reliance on psychological preservation tactics—alternating between conscious avoidance, spiritual surrender to a higher power, and resilient future-oriented aspirations—to reclaim a sense of agency and humanity from a demanding, lifelong pathology.

### Themes

In this study, 4 main themes were identified, with several subthemes underneath each theme. These include psychoemotional factors, supportive factors, challenges to DM management and wellness, and coping mechanisms (Table 2).

**Table 2.** Summary of themes, subthemes, and codes of adolescent experiences living with type 1 diabetes mellitus (T1DM) who have a follow-up at Felege Hiwot Hospital, Bahir Dar, Ethiopia, 2023.

Themes	Theme 1: psychoemotional factors	Theme 2: supportive factors	Theme 3: challenge to DM^a^ management and well-being	Theme 4: coping toward negative psychoemotional factors
Subthemes	Psychological factors Negative Emotional factors Positive emotional factors	Community-Based Health insurance (CBHI) Hospital care and support Supportive school environment Good family and friend support	Challenge in adherence A challenge to get insulin Challenge to sleep	Avoidance coping, acceptance coping, and adopting coping Social coping Spiritual coping
Codes	Stress Anxiety Feeling of inferiority Low self-esteem Sense of being different from others Hate Sadness Anger Heartbreak Frustration Awfulness Love Happiness Hopefulness Hopelessness Fear Shame	Financial support Friend knowledge of symptoms and management Empathy Advice Help Respect Safe place Family love Psychological support Create a feeling of equality Excellent care Satisfaction	Fasting Sleepy Difficult to awake Difficult to initiate sleep Hypoglycemia Insulin unavailability Insulin inaccessibility	Ignore Sleeping Hope Accepting Adopt it Draw picture Watch movies Positive self-talk Seeking help Prayer

a DM: diabetes mellitus.

### Theme 1: Psychoemotional Factors

#### Psychological Factors

Most adolescents living with T1DM revealed psychological factors, including stress, anxiety, feelings of inferiority, low self-esteem, and a sense of being different. Adolescents who struggled with these issues found it difficult to function in their daily lives and experienced a range of problems. All of these factors made it harder for adolescents with type 1 diabetes to engage in normal activities, social life, and school performance. This led to negative impacts on their overall well-being, which could even exacerbate their physical symptoms or contribute to the emergence of other problems such as depression. Addressing psychological factors and promoting self-acceptance can improve the well-being of adolescents with T1DM.

These problems stem from the nature of the disease, the burden of insulin injections, and the lifelong need to manage blood sugar levels. This constant medical regimen contributed to the adolescent feeling inferior. For example,

*I have come to realize that my care is continuous and lifelong; I always need to follow up with appointments at the hospital and take medication to stay alive. This situation makes me feel disgusted, and when I think about it, I feel bad and inferior.*

[ 17-year-old, female]

Another psychological issue concerned how participants perceived themselves as patients. Many participants described defining themselves primarily as patients, which can lead to feelings of dependence and low self-worth. This perception can affect their mental health and quality of life.

*…I always feel inferior when I think about having a disease. What I couldn’t stop dwelling on, and what caused me pain, was the belief that I am inferior because of my diabetes.*

[An 18-year-old, male participant]

#### Negative Emotional Factors

The study revealed that individuals living with diabetes experience negative emotions, including anger, frustration, embarrassment, fear, hopelessness, heartbreak, and shame. Many participants expressed feelings of sadness and questioned why they had this disease. Some felt self-conscious about administering their insulin injections in front of others and avoided doing so in public. Family members were frustrated and distressed when observing their child’s daily insulin injections. The fear of complications such as blindness, hypoglycemia, and microvascular and macrovascular damages were common concerns that led to negative emotions.

*I am always questioning why I’m living with DM, what I did wrong and why God gave me this disease. This make me feel bad ... Sometimes I’m scared, especially when I consider potential complications like blindness. living with DM, I may experience life threatening accidents caused by falling down due to hypoglycemia. This always frustrates me.*

[A 15-year-old, female adolescent]

#### Positive Emotional Factors

In the study, some adolescents shared positive emotions such as happiness and optimism about their future, which were not defined by their illnesses but by the quality time spent with family and loved ones. They expressed feeling healthy and content when enjoying moments together. One 17-year-old female participant said, “I’m very happy spending time with my family and friends at recreational places, and I feel just as healthy with them.”

### Theme 2: Supportive Factors

#### CBHI

Based on the findings of this study, CBHI proved to be a valuable support system for adolescents with T1DM. Almost all participants expressed gratitude for the financial protection provided by CBHI, which helps to minimize the burden of paying out-of-pocket expenses. They also desired coverage of medical costs incurred outside of the country.

*I don’t pay for medicine, only for transportation, thanks to our family health insurance. Without it, medical expenses would be a struggle for both to me and my family. I believe having health insurance is one of the reasons why I can continue to live here.*

[A 17-year-old, female participant]

Similarly, a 16-year-old male adolescent stated, *“*Health insurance is beneficial, but it would be even more helpful if it could provide financial assistance to Ethiopians in need of medical treatment abroad.*”*

#### Hospital Care and Support

Most participants expressed that the care and counseling provided by health care providers was of high quality. The patients were satisfied with the care and support provided in the facility. The hospital personnel also corrected any mistakes made while injecting insulin and appreciated the patients when they got it right, which helped build trust and confidence in their self-care management.

A 13-year-old female participant said, *“*I received exceptional care at the facility, which ultimately prevented me from death. The quality of instruction and the support were excellent*.”*

*The follow-up care at this hospital is excellent, but I also received good care and follow-up at my previous hospital. The staff in this facility are attentive and inquire about my insulin injection technique, correcting any mistakes and acknowledging correct procedures. They also provide education on proper diabetes management, including warnings against consuming sweet foods*.

[A 16-year-old, female participant]

#### Good Family, Friends, and Community Support

Most participants expressed that they have good support from family members, friends, and the community. The family members of adolescents are supportive in reminding them to take insulin, taking necessary precautions, and actively helping them when they are sick. Similarly, their friends play a significant role in supporting them when they are feeling bad and need academic support. Friends help keep them up-to-date with schoolwork and inform teachers about any problems. For example,

…*My friends love me like their brothers and sisters, and I am love them too. For example, when I have an appointment at the hospital, they pay close attention in class and update me on what I missed. They inform the teacher if I’m having difficulties and act to support me. I never feel alone because they are always with me. My father reminds me when it’s time to take my medicine, just like my mother did. They do everything possible for me when I’m sick. Even my sister is there for me....*

[A 17-year-old, female participant]

Furthermore, some adolescents mentioned that they received financial support from their relatives and neighbors, which helped them pay for their treatment and made it possible for them to see this day. A 16-year-old female said*, “*… What’s surprising is I’m here now because my neighbor supported me financially at the beginning; I didn’t have enough money at that time.”

#### Supportive School Environments

Most of the participants expressed that the school environment was supportive of adolescents living with T1DM. They stated that they felt safe at school, with their classmates and teachers showing empathy toward their conditions. Moreover, almost all participants mentioned that they did not experience any health-related issues at school, which could be attributed to the supportive environment. For example, a 15-year-old male participant said, *“*There are no challenges for adolescents living with diabetes to attend school and I feel safe and healthy.”

Another 13-year-old female said*, “*…both my classmates and teachers always support me when I need help” (P6).

Additionally, some adolescents with type 1 diabetes have disclosed their illness to friends at school and feel comfortable talking about it. They have developed close relationships with both male and female classmates and feel positive about their school experience. They have also noted that people at school want to help them, which suggests a supportive culture around chronic illness.

*I have a good relationship with my classmate, and they are aware of my condition. The school atmosphere is welcoming, and everyone is willing to help me*.

[A 16-year-old, male participant]

### Theme 3: Challenges to DM Management and Well-Being

#### Challenges Toward Adherence

Even though all participants reported adhering to their medication most of the time, they faced challenges in doing so. While fasting was not mandatory, some participants attempted it, viewing it as harmless. They fasted without taking their morning injections, which highlighted how fasting could challenge their adherence to insulin.

A 16-year-old female participant shared, “During fasting season, I have never taken my morning medication. I assumed it wouldn’t harm me, so I continued fasting.”

Some participants mentioned missing doses when traveling without their insulin. An 18-year-old female participant said, *“*Sometimes I forget my medication when I travel to places like holy water without my insulin…”

Moreover, some participants struggled with adhering to their insulin injections due to the fear of hypoglycemia from demanding activities. This fear stemmed from the severe side effects of hypoglycemia, such as dizziness, weakness, and heart palpitations, which can be alarming. Consequently, they might avoid injecting insulin during extended travel or activities that could trigger hypoglycemia. For example,

*If I’m traveling for an hour, I skip my insulin because injecting it before or during long journeys could cause a sudden drop in blood sugar levels, leading to dizziness and heart palpitations, which is scary.*

[A 17-year-old female participant]

Some participants also mentioned adjusting their medication schedules to accommodate their study routines. An 18-year-old male participant stated, *“*I sometimes delay my nighttime dose to match my study schedule.”

#### Challenges to Getting Insulin

Most of the participants living with T1DM expressed concerns about the unavailability and inaccessibility of insulin. This had serious consequences for individuals with diabetes, especially during crises such as the COVID-19 pandemic. The lack of access to insulin made it challenging for individuals to effectively manage their diabetes, resulting in various negative health outcomes.

Furthermore, the financial burden of obtaining insulin in areas where it is available can be significant, adding to the challenges faced by individuals with diabetes.

*The most pressing issue is the availability of insulin, which is a major challenge. The Corona period was very difficult for us because we struggled without insulin.*

[An 18-year-old, male adolescent]

*I had planned to seek medical treatment at X Hospital. However, the hospital staff informed me that they did not currently have insulin available, making it hard for me to receive treatment there. This situation imposes a significant financial burden on me, as I typically spend around 300 birrs on transportation alone, in addition to other expenses like food. If insulin were available at that hospital, it would greatly reduce the financial strain I am currently experiencing.*

[A 15-year-old, male participant]

#### Challenges to Sleeping

Almost all of the adolescent participants living with type 1 diabetes experience sleep problems, such as sleepiness, difficulty waking up in the morning, and trouble initiating sleep. These issues can exacerbate their emotional challenges, affecting their overall well-being. The sleep problems are often caused by the physical symptoms of diabetes, such as frequent urination, thirst, and hunger, as well as the emotional stress of the disease. Lack of sleep can lead to irritability and difficulty concentrating, making it harder to maintain a healthy lifestyle. An 18-year-old female said*, “*I sleep too much, which makes me feel lethargic. Sometimes, waking up in the morning is difficult for me…”

*I experience difficulty falling asleep because of unidentifiable concerns that run through my mind. I feel unable to talk about this matter with anyone.*

[A 16-year-old, male participant]

### Theme 4: Coping Toward Negative Psychoemotional Factors

#### Adaptation, Acceptance, and Avoidance Coping

Most participants mentioned avoidance, adoption, and acceptance as major coping methods for their psychoemotional problems. Some actively choose to adopt a positive and hopeful mindset through self-talk. For example, a 13-year-old female participant envisioned a future career as a scientist making diabetes medicine, stating, *“*…I tell myself that I want to be a scientist in the future and develop medicine for diabetes whenever I experience psychoemotional problems.”

Others mentioned accepting their circumstances and limitations, recognizing that some things were beyond their control. Participants expressed understanding and a sense of peace regarding their situation, allowing them to move forward without worry. A 16-year-old female participant said, “I understand that it’s not my fault. I have given it to the creator, as he knows about his gift, and now I don’t have to worry about it.”

On the other hand, some participants choose to avoid or ignore their problems by sleeping or engaging in distractions like drawing or watching a movie. A 17-year-old female participant mentioned, *“*When I feel angry or experience any negative emotions, I cope by drawing a picture and watching a movie.”

Similarly, 15-year-old female participants said, *“*When I experience negative emotions or stress, I tend to ignore them and go to sleep.”

#### Social Coping Mechanism

Nearly half of the participants use social coping strategies as a means of dealing with their psychoemotional problems. These coping mechanisms include seeking support from friends and family, consulting with trusted individuals, and engaging in activities with others. This participant focused on building relationships and finding comfort in the company of others, rather than trying to solve the problem alone. This is supported by:

*If I am experiencing stress, anxiety, anger, fear or any other negative emotions, I find it helpful to spend time with my friends to relax and feel better. Together, we might take a shower, engage in activities, and ultimately alleviate the negative emotions I am experiencing.*

[A 15-year-old, male participant]

Additionally, an 18-year-old female said*, “*If I’m experiencing any emotional instability, I find that talking or playing with my friends, makes me feel good.*”*

#### Spiritual Coping

Some participants explained that prayer helped them relive emotional distress and use it as a coping mechanism. A 16-year-old male participant expressed his experience as, “… if I have any negative emotions, I will go to the church and pray to the God for peace and guidance.*”*

**Figure 1.**
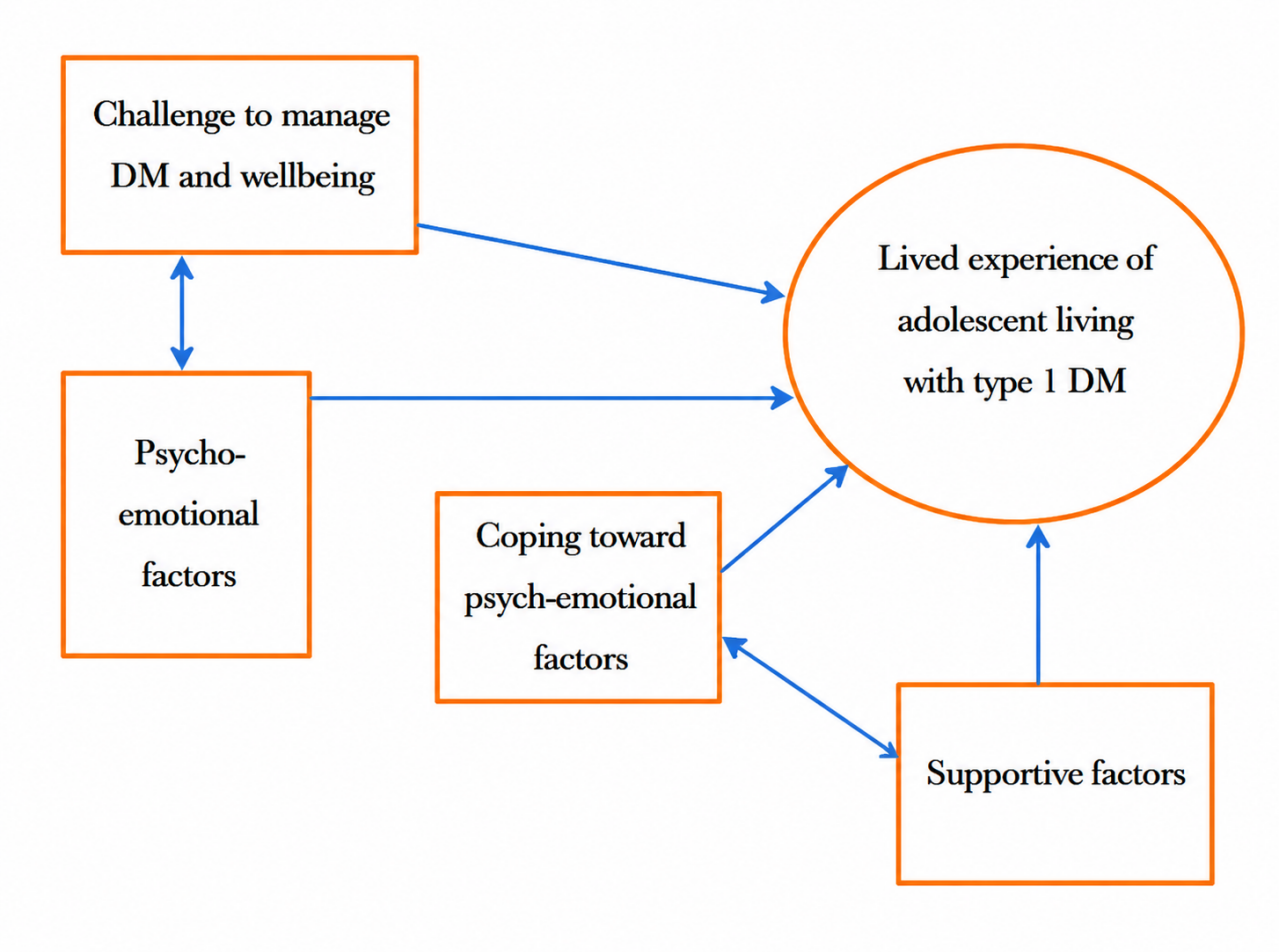
A model that shows how each topic in the overall lived experiences of teenagers connects to the others. DM: diabetes mellitus.

The results showed that the themes and their subthemes had a clear link. As a result, researchers developed a model that shows how each topic in the overall lived experiences of teenagers connects to the others (Figure 1)

## Discussion

### Principal Findings

Understanding the lived experience of adolescents living with T1DM from their own perspective is a crucial foundation for improving care, particularly in low-resource settings where social, economic, and health-system constraints shape daily disease management. This phenomenological study explored the lived experiences of adolescents with T1DM in Bahir Dar, Ethiopia, a context that is rarely represented in the global diabetes literature. Four interrelated themes emerged, including psychoemotional experiences, supportive factors, challenges to diabetes management and well-being, and coping with negative psychoemotional factors. Together, these themes provide insight into how adolescents navigate T1DM within a setting characterized by limited resources, strong family and community ties, and variable access to specialized diabetes care.

The findings of this study reveal that adolescents living with T1DM experience feelings of inferiority and a sense of being different from their peers, often perceiving themselves as inadequate or less capable in managing their condition. These perceptions may undermine self-esteem and negatively influence both social participation and self-management behaviors. Similar findings have been reported in previous studies, which show that adolescents with type 1 diabetes frequently experience social isolation due to physical limitations, the visibility of treatment routines, and stigma associated with their condition, leading to feelings of exclusion or rejection by peers [29]. In addition, participants described a wide range of emotional experiences, including sadness, frustration, heartbreak, and emotional exhaustion, reflecting the psychological burden of living with a chronic condition. The demanding and continuous nature of diabetes self-management—such as insulin administration, blood glucose monitoring, and dietary regulation—can be disruptive to daily life and contribute to emotional distress. These challenges may be further intensified during adolescence, a developmental stage marked by hormonal changes, identity formation, and heightened sensitivity to peer acceptance. Consistent with previous studies, adolescents with type 1 diabetes reported difficulties related to treatment demands, negative perceptions of diabetes, school-related challenges, and uncertainty about the future, all of which can contribute to negative emotional and psychological outcomes [18]. These findings underscore the importance of integrating psychosocial support, emotional counseling, and peer-based interventions into routine diabetes care to address both the emotional and social dimensions of living with type 1 diabetes and to support adolescents in developing resilience and effective coping strategies.

Several supportive factors were identified that may contribute to good glycemic control, improved treatment adherence, effective adjustment to chronic conditions, and enhanced quality of life among individuals living with chronic illnesses. One important supportive factor identified in this study is CBHI, which plays a significant role in assisting individuals to manage long-term conditions such as diabetes. This support is particularly crucial for patients who require continuous medical care, regular follow-up, and lifelong treatment. By reducing financial barriers, CBHI facilitates access to essential health care services and medications that might otherwise be unaffordable, thereby improving continuity and quality of care. Improved access to services through CBHI may also promote better adherence to treatment and more consistent disease monitoring, which are essential for optimal health outcomes. These findings are consistent with evidence from Ethiopia, where studies have reported high levels of patient satisfaction with the overall quality of health care services provided through CBHI, highlighting its potential to strengthen chronic disease management and improve patient outcomes [30].

This study shows that adolescents receive adequate support from family, peers, friends, and schools, which is identified as a supportive factor for disease control and quality of life. Adolescents with diabetes encounter numerous challenges, including managing blood sugar levels, addressing the stigma associated with the condition, and coping with the emotional stresses of living with a chronic illness. Therefore, support from family, community, peers, and school can significantly assist in helping adolescents manage their condition and improve quality of life. A study indicates that greater satisfaction with diabetes self-management support from schools, family, peers, and friends is linked to improved metabolic levels and quality of life in adolescents with T1DM [25]. Our finding contradicts that of another study conducted in Ethiopia, which found difficult family dynamics to support the care [25]. Therefore, the presence of a supportive family is important to improve their health conditions and quality of life.

The findings of this study indicate that adolescents living with type 1 diabetes face significant challenges related to the unavailability and inaccessibility of insulin, particularly during crises such as the COVID-19 pandemic, which negatively affected diabetes management and health outcomes. These findings are consistent with global evidence showing that access to insulin remains limited due to high prices, supply-chain markups, weak national procurement and distribution systems, and market dominance by a small number of multinational manufacturers [31,32]. Despite insulin being essential for survival in type 1 diabetes, insufficient policy attention, inadequate health system preparedness, and lack of financial protection continue to hinder equitable access, especially in low- and middle-income settings [33]. The experiences reported by adolescents in this study, therefore, reflect broader global and systemic failures in ensuring reliable access to insulin, underscoring the urgent need for coordinated policy and health system interventions to protect vulnerable populations during both routine care and public health emergencies.

Another important factor influencing glycemic control among adolescents with T1DM is poor treatment adherence, which may result from competing demands such as school workload, travel, or participation in religious fasting. Fasting carries profound spiritual significance for individuals observing religious practices; however, for adolescents with type 1 diabetes, it can pose serious risks to maintaining stable blood glucose levels. While some studies suggest that adolescents with good glycemic control under close medical supervision may fast safely during Ramadan [34], there is limited research on the physiological effects of Christian fasting on anthropometric and biochemical parameters [35]. The findings of this study highlight that fasting can disrupt treatment adherence and glycemic control, potentially endangering the health of adolescents with type 1 diabetes. These results underscore the need for culturally sensitive education and counseling, enabling adolescents to understand the risks of fasting, implement safe management strategies, and maintain consistent adherence to insulin therapy and self-care routines, even during religious observances.

Our study shows that sleeping disturbances and lack of well-being are common problems faced by adolescents living with T1DM. They experience difficulty falling asleep, staying awake, and increased sleeplessness. Other studies have also shown that adolescents with DM often experience sleeping disturbances [19,36]. Fluctuations in blood sugar levels can lead to nocturnal hypoglycemia or hyperglycemia, disrupting sleep [24]. Furthermore, the stress and anxiety related to manage the illness can also contribute to sleep disturbances [34]. This finding suggests that health care providers should consider the impact of sleep on glycemic control.

This study reveals that the participants used 3 main coping mechanisms, including adaptation, acceptance, and avoidance coping, including social and spiritual coping mechanisms. This is consistent with findings from studies conducted in Zambia, indicating that individuals facing chronic illnesses like diabetes may have shared coping experiences [26]. Taken together, the results highlight the importance of addressing the emotional and psychological needs of adolescents with type 1 diabetes through a holistic approach that incorporates personal, social, and spiritual dimensions, alongside medical care, to support resilience, adherence, and overall quality of life.

This study has several limitations that should be considered. First, it was conducted in a single hospital setting, which may limit the transferability of findings to other regions or health care environments. To mitigate this, researchers spent extended time in the field, built rapport with participants, and provided rich contextual descriptions. Future studies could include multiple sites to capture broader perspectives. Second, the sample size was relatively small, comprising 10 participants selected through purposive sampling. Although data saturation was achieved, the limited number may not fully reflect the diversity of adolescent experiences across Ethiopia. Maximum variation sampling was used to include participants of different ages, genders, residences, and duration of illness, but larger, more diverse samples could strengthen generalizability in future research. Third, additional clinical and contextual variables, such as comorbidity, type of insulin, blood glucose monitoring methods, hemoglobin A_1c_ (HbA_1c_) levels, BMI, and family history, were not collected, though they could provide deeper insight into adolescents’ experiences with T1DM. Despite these limitations, the study contributes meaningfully to adolescent-centered diabetes care and highlights the importance of emotional well-being, consistent insulin access, and culturally sensitive management strategies.

### Conclusion

This study captures the holistic essence of adolescents with T1DM at Bahir Dar, Ethiopia, exposing a reality where clinical management is inextricably bound to psychological survival and socioeconomic vulnerability. This study makes a unique contribution to the global phenomenology of chronic illness by shifting the narrative away from high-resource Western contexts to showcase how adolescent identity negotiation occurs within the unique cultural, economic, and structural realities of regional Ethiopia. It demonstrates that the financial protection of CBHI and strong collective social networks are vital; yet, they remain vulnerable to systemic failures like medication supply-chain collapses. There is an urgent policy and clinical implication to establish dedicated, multidisciplinary adolescent endocrine clinics within regional referral hospitals. These specialized spaces must formally integrate routine mental health screenings, peer-led support networks, and localized nutritional counseling into the standard monthly follow-up track, transforming diabetes care from an episodic medical intervention into a comprehensive, youth-centered developmental support system.

## Supplementary material

10.2196/71179 Multimedia Appendix 1 The complete semistructured interview guide used in this study.

10.2196/71179 Checklist 1 SRQR checklist.

## Abbreviations

CBHI: Community-Based Health Insurance
DM: diabetes mellitus
FHCSH: Felege Hiwot Comprehensive Specialized Hospital
HbA_1c_: hemoglobin A_1c_
IRB: Institutional Review Board
PI: principal investigator
T1DM: type 1 diabetes mellitus
WHO: World Health Organization
