# Supplementary material for: The Lived Experience of Adolescents With Type 1 Diabetes Mellitus in Bahir Dar, Ethiopia: Phenomenological Study
Source: JMIR Diabetes. 2026 Aug 5;11:e71179. doi: 10.2196/71179 (PMC13448013; doi:10.2196/71179)
Supplement: Supplementary file 1 [file diabetes-v11-e71179-s002.pdf]

Completed SRQR Checklist

Manuscript Title: *The lived experience of adolescents with type 1 diabetes mellitus in Bahir Dar, Ethiopia: a phenomenological study*

SRQR Item	Description	Reported on Page No.
1. Title	Concise description of the nature and topic of the study; identifies the study as qualitative or indicates the approach	Title page
2. Abstract	Summary of key elements of the study using the abstract format of the intended publication	pp. ii–iii
3. Problem Formulation	Description and significance of the problem/phenomenon studied; review of relevant theory and empirical work	pp. 4–5
4. Purpose or Research Question	Purpose of the study and specific objectives or questions	p. 5
5. Qualitative Approach and Research Paradigm	Descriptive phenomenological design and Husserlian phenomenology with rationale	pp. 6, 8–9
6. Researcher Characteristics and Reflexivity	Researcher background, reflexivity, and bracketing (epoche)	pp. 8–9
7. Context	Study setting and contextual information about the hospital and diabetes clinic	p. 6
8. Sampling Strategy	Purposive sampling,	pp. 6–7

SRQR Item	Description	Reported on Page No.
	eligibility criteria, recruitment process, and data saturation	
9. Ethical Issues Pertaining to Human Subjects	IRB approval, informed consent/assent, confidentiality, and participant rights	pp. 9–10, 23
10. Data Collection Methods	Face-to-face in-depth interviews, interview procedures, recording, and follow-up	pp. 7–8
11. Data Collection Instruments and Technologies	Semi-structured interview guide, audio recorder, ATLAS.ti software	pp. 7–9
12. Units of Study	Number and characteristics of participants	pp. 10–11
13. Data Processing	Verbatim transcription, translation, coding, anonymization, and storage	pp. 8–10
14. Data Analysis	Colaizzi's seven-step phenomenological analysis and thematic development	pp. 8–9
15. Techniques to Enhance Trustworthiness	Credibility, dependability, confirmability, transferability, member checking, audit trail	pp. 7–8
16. Synthesis and Interpretation	Themes, subthemes, essence of lived experience, and interpretation	pp. 11–22
17. Links to Empirical Data	Direct participant quotations supporting findings	pp. 12–18

SRQR Item	Description	Reported on Page No.
18. Integration with Prior Work, Implications, Transferability, and Contributions	Discussion of findings in relation to previous studies and implications for practice/policy	pp. 18–23
19. Limitations	Study limitations and recommendations for future research	p. 22
20. Conflicts of Interest	No conflicts reported in manuscript	p. 24
21. Funding	Funding statement indicating no financial support received	p. 24
