# Supplementary material for: The Lived Experience of Adolescents With Type 1 Diabetes Mellitus in Bahir Dar, Ethiopia: Phenomenological Study
Source: JMIR Diabetes. 2026 Aug 5;11:e71179. doi: 10.2196/71179 (PMC13448013; doi:10.2196/71179)
Supplement: Supplementary file 2 [file diabetes-v11-e71179-s001.docx]

**DEMOGRAPHIC INFORMATION QUESTIONNAIRE (English version)**

Can you please tell me some identifying information about yourself? Yes	no

**Code;**

1. How old are you?
2. Are you male or female?
3. Where do you live?
4. What grade are you in?
5. How long have you been living with diabetes?
6. With whom do you live?
7. Do you have health insurance?

**SEMI-STRUCTURED IN-DEPTH INTERVIEW GUIDE**

1. **Can you tell me about your life since you were diagnosed with type 1 diabetes?**
2. **How does having diabetes affect how you see yourself and how you feel about your life?**
3. **What kinds of feelings or thoughts do you usually have because of living with diabetes?**
4. **Can you describe what it is like for you to manage insulin, food, school, and daily activities?**
5. **What helps you most to live with diabetes and continue your daily life?**
6. **What are the biggest difficulties you face because of your diabetes?**
7. **How does your family, friends, school, or community treat and support you?**
8. **When you feel worried, sad, or stressed about diabetes, what do you do to cope?**
9. **Is there anything else about your experience with diabetes that you would like to share?**
